# Dynamic Monitoring of Time-Dependent Evolution of Biomolecules Using Quantum Dots-Based Biosensors Assemblies

**DOI:** 10.3390/bios14080380

**Published:** 2024-08-07

**Authors:** Razvan Bocu

**Affiliations:** Department of Mathematics and Computer Science, Transilvania University of Brasov, 500036 Brașov, Romania; razvan.bocu@unitbv.ro; Tel.: +40-732011010

**Keywords:** biosensors, electrochemical biosensors, quantum dots biosensors, biomolecules, cell membranes

## Abstract

The dynamic monitoring of biomolecules that are part of cell membranes generally constitutes a challenge. Electrochemiluminescence (ECL) biosensor assemblies provide clear advantages concerning microscopic imaging. Therefore, this paper proposes and analyzes a quantum dots-based biosensor assembly. Thus, particular attention is granted to biomolecules that are part of cell membranes. Additionally, this paper describes and analyzes a quantum dots-based biosensor assembly, which is used to implement a fully functional color ECL visualization system that allows for cellular and biomolecular structures to be accurately visualized. The related nano-emitter allows the implementation of real-time bioimaging scenarios. Consequently, the proposed approach is thoroughly evaluated relative to the time-dependent evolution of biomolecules. It has been demonstrated that traditionally problematic structures, like the biomolecules that are part of cell membranes, can be studied and monitored relative to their time-dependent dynamic evolution using the proposed solution. The reported research process has been conducted in the realm of cooperation with a specialized biomedical engineering company, and the described results are expected to substantially support a better understanding of the biomolecules’ time-dependent dynamic evolution.

## 1. Introduction

The proper placement of membrane proteins relative to the cell surface is biologically regulated. This has the role of providing the required level of stable cellular function [1,2,3]. As a consequence, it is essentially mandatory to comprehend the dynamic features of the biomolecules’ motion relative to the membranes of cells. The tracking of individual particles (TIP) represents a reliable functional modality, which may support the proper assessment of the individual particles’ functional features relative to the cell membranes [4,5]. It is relevant to note that the design of several fluorescent elements mediates the consideration of fluorescent TIP tracking processes for the analysis of dynamic molecular processes, such as molecular interactions and lateral diffusion events [6,7,8]. More precisely, the dynamic evolution of an individual molecule that is marked through a fluorescent modality relative to a particular biological cell may be tracked during a sufficiently long time span, in a real-time manner. The precise generation of the cell’s motion patterns also allows the acquisition of technical information regarding the physical features of cells, the dynamic modalities of cell membranes, and the intracellular motion patterns.

Nevertheless, certain support surfaces, such as fluorescent proteins and organic dyes, provide an insufficient fluorescent signal, and also below average photostability. Therefore, it is necessary to consider high power laser devices to implement TIP tracking processes. Unfortunately, this approach produces high optical background signals and substantially reduces the possibility of applying fluorescent TIP techniques for the continued tracking of the biomolecules through a multiple-color process [9,10]. Consequently, it is immediate to note that non-invasive and very accurate bioimaging and monitoring techniques of single molecules are necessary.

Electrochemiluminescence (ECL) effects are determined by electrochemically sensitive components, which can be efficiently controlled and determined by a low background noise signal. Consequently, imaging approaches that are based on ECL processes may be precise and time efficient [11,12]. Additionally, they may be deployed through the consideration of plain real-world optical setups [13]. Considering the emergent nature of this technical approach, biomolecular imaging that is based on ECL processes has been effectively considered to evaluate the motility of individual cells. Additionally, this may also approach the morphological modifications of cells and the distribution of proteins in the cell membranes [14,15]. Nevertheless, the real-time visual analysis of individual biomolecules [16] at the level of the cell membranes through ECL imaging processes represents a complicated endeavor due to the low-power ECL signals generated by certain nano-emitters [17,18]. As a consequence, particular recent nano-emitters that feature higher-power ECL signals and superior stability may be considered labels for the respective ECL signals. This approach may also allow the implementation of color visualizations relative to the target cell membranes.

Semiconductor quantum dots (SQD) feature certain photophysical features, like improved brightness and emission bandwidths that pertain to narrow color emissions. Consequently, these may be considered to be viable solutions for the implementation of the required nano-emitters regarding TIP tracking processes, which consider electrochemiluminescent signals [19,20,21,22]. The augmentation of the respective ECL signals, which relate to the SQDs, involves that proper reactants may be considered to provide the necessary electrons [23,24]. Considering that hydrogen peroxide (H_2_O_2_) is a fundamental constituent of reactive oxygen molecules, it may be regarded as a reactant to improve the power levels of the ECL signals that are featured by the quantum dots (QD) [25,26]. Nevertheless, the presence of hydrogen peroxide is fundamentally related to the state and type of cells [27,28] that are involved in the experimental processes. Therefore, hydrogen peroxide may not be considered to be a reliable generator of reactants relative to long-term real-time ECL imaging processes. Consequently, the research evaluation that was conducted arrived at the conclusion that electrochemiluminescence processes, which are based on quantum dots, may be considered for the implementation of the required biomolecular TIP tracking processes.

**Figure 1:**
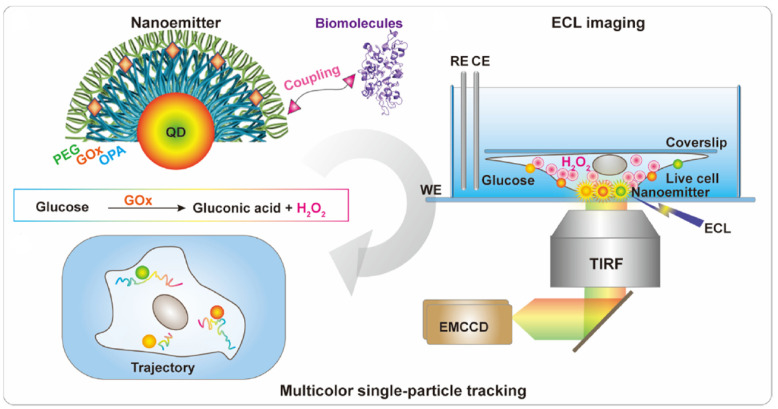
Implementation of the biomolecular TIP tracking processes using quantum dot electrochemiluminescent emitters. The figure displays the preparation of the QD-based nano-emitters, the color nano-emitters considered for ECL imaging on cell membranes, and the trajectories of biomolecules monitored through the ECL color TIP tracking process.

The applied research process that is reported in this paper considers the design of an electrochemiluminescent quantum dot nano-emitter. This is selected to design and implement the required efficient color biomolecular TIP tracking processes relative to the cell membranes. Thus, the ECL nano-emitter considers two fundamental components, which are CdZnSeS quantum dots and glucose oxidase (GOx). The quantum dots emit the ECL signals, and GOx mediates the processing of environmental glucose to generate the related reactant hydrogen peroxide. Thus, the logical and functional features of this process are represented in Figure 1.

Following, it is relevant to describe certain technical constraints and solutions, which were considered and effectively applied. The construction of the ECL emitters relates to the water-dispersible quantum dots, which were generated through the modification of CdZnSeS quantum dots through the addition of octylamine-conjugated polyacrylic acid. The resulting reactive place mediates the proper binding between molecules through the consideration of carboxyl-amine reactions. The obtained ECL emitters possess several relevant features. Thus, the enhancement of the compound with glucose oxidase circumvents the occurrence of blanks or artifacts during the biomolecular imaging processes. Furthermore, the considered quantum dot hydrogen peroxide ECL solution offers the possibility to implement the necessary real-time bioimaging processes relative to the target cell membranes. Additionally, the experimental assessment demonstrated that the approach generates a sufficiently high signal-to-noise ratio (SNR). Additionally, it is relevant to note that the color TIP tracking processes may be implemented to isolate the biomolecular interactions, which occur at the level of the cell membranes. Therefore, the experimental assessment that was conducted demonstrated that ECL emitters may be considered to design and implement the required ECL TIP tracking processes.

The integrated monitoring and visualization system that is proposed features a series of unique features relative to semantically similar approaches that are reported in the reviewed scientific literature. Thus, it considers readily available organic, inorganic, electrical, and electronic components to design and implement the required infrastructure. It can accurately detect the motion of biomolecules on the target cells’ surface. Although the experimental evaluation process considers the membranes of the A549 cells, the integrated approach may be considered to monitor and visualize the biomolecules that are attached to any cell. Consequently, it precisely monitors the time-dependent evolution of the biomolecules that exist on the target cells’ surface. It generates consistently sharp and clear image frames over time, and it can produce color images while the respective color details are rendered precisely in accordance with their biomolecular role. It is important to note that the generated image frames’ resolution is sufficient to precisely capture the motion of the analyzed biomolecules. Consequently, it is possible to accurately represent the paths of the observed biomolecules on the surface of the respective cells, both considering experimental laboratory settings and real-world use case scenarios.

## 2. Materials and Methods

The experimental process that is described was organized in the laboratory that was provided by the partner biomedical company.

### 2.1. Design of the ECL Emitters

The initial stage of the experimental process considered wheat germ agglutinin (WGA), which is used to discover N-acetylglucosamine and sialic acid molecules on the monitored cell surfaces [29,30]. This is considered to be an experimental reference molecule, which evaluates the appropriateness of the designed emitter.

Following, the CdZnSeS quantum dots, which are used to implement the color TIP bioimaging processes, considered several wavelengths during the experimental calibration process, which validated 540 nm, 585 nm, and 630 nm as possible wavelengths for the experimental functional solution. As an example, considering the quantum dots relative to a wavelength of 540 nm, it may be asserted that they feature a uniform size of 9 nm, a high quantum yield at 96%, and a sufficiently long lifespan of the fluorescent elements of 52 ns.

**Figure 2:**
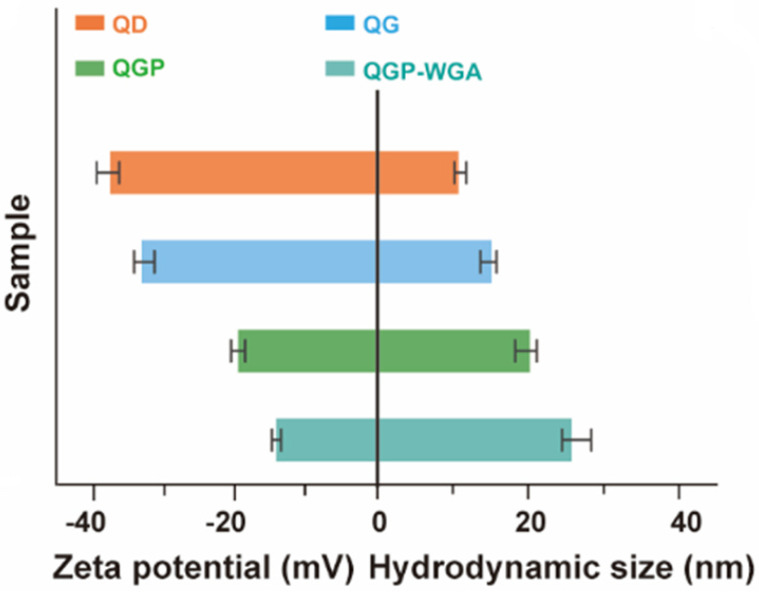
Representation of the obtained Zeta potential and related hydrodynamic size. The Zeta potential is represented in the left half of the graph, while hydrodynamic size relates to the right half of the graph.

Consequently, the experimental calibration considered a dynamic light scattering process and also zeta potential measurements in order to monitor the construction flow of the ECL emitter. It is relevant to note that the zeta potential measurements assess the difference in potential between the bulk fluid through which a particle is dispersed and the layer of fluid that includes the oppositely electrically charged ions, which relates to the target nanoparticle’s surface. Moreover, the size of the nanoparticles was successively increased, and the potential eventually turned to be positive, considering an incremental fine-tuning process. Essentially, this outcome formally suggested that the design solution of the emitter reached the optimal variant, as is also suggested by Figure 2.

The CdZnSeS quantum dots were manufactured considering the approach that is known as the hot-injection technique. Considering the first step, a compound made of 1.3 g of CdO, 0.6 g of hexadecylamine (HAD), 50 mL of octadecene (ODE), and 30 mL of oleic acid (OA) was loaded into a three-neck flask, which was stirred and heated to 280 °C under $N_{2}$. As soon as the temperature of the solution neared 260 °C, trioctylphosphine (TOP) (2.23 mL) and Se/TOP (12 mL) precursors were added to the Cd-HAD-OA solution to commence the nucleation and growth of the binary CdSe seeds. Moreover, samples of Se/TOP (12 mL), ZnO/OA (20 mL), and S/trioctylphosphine oxide (TOPO) (50 mL) were added into the specified compound solution to generate the nucleation and growth of the CdZnSeS quantum dots. The generated quantum dots, which are part of the obtained raw solution, were isolated considering a centrifugation process.

It is relevant to note that, considering the validation of the quantum dots generation process, the CdZnSeS quantum dots were monitored using transmission electron microscopy (TEM) relative to a Talos F200X G2 system manufactured by Thermo Fisher Scientific in Waltham, MA, USA. The data regarding the absorption spectra were recorded at room temperature using a V-770 Double Beam UV-Visible/NIR Spectrophotometer, which is manufactured by JASCO in Oklahoma City, OK, USA. It is relevant to note that the PL spectral and PL delay curves were quantitatively evaluated relative to the FLS1000 spectrometer, which is manufactured by Edinburgh Instruments (Livingston, UK). The temperature-dependent precalibration processes were conducted using a FluoroMax Plus Spectrofluorometer, which is manufactured by Horiba Scientific (Piscataway, NJ, USA).

Following, the experimental evaluation process assessed the effective performance of the quantum dot elements. The quantitative ECL evaluation (measurements) was designed using three electrodes. These include a reference electrode (Ag/AgCl), a counter electrode made of platinum wire, and a working electrode based on indium tin oxide. Initially, the experimental flow detected no meaningful levels of ECL signals emitted by the quantum dots. Furthermore, the addition of hydrogen peroxide (2.8 mM) determined the generation of a significantly enhanced ECL signal by the quantum dots. It is also relevant to note that the addition of 0.68 μM of glucose did not determine an enhancement of the emitted ECL signal at the level of the quantum dots. Furthermore, the experimental setup also evaluated the variation of the ECL signal intensity relative to the apparent increase of time intervals in the cell culture medium. Thus, the measured signal was relatively constant, which demonstrates a proper level of stability in the ECL environment.

Overall, the experimental results demonstrate that the ECL emitter, which was properly changed with GOx, may produce reactants relative to certain cell culture conditions. The experiments that were conducted prove that this essentially mediates the implementation of the required real-time ECL bioimaging process.

**Figure 3:**
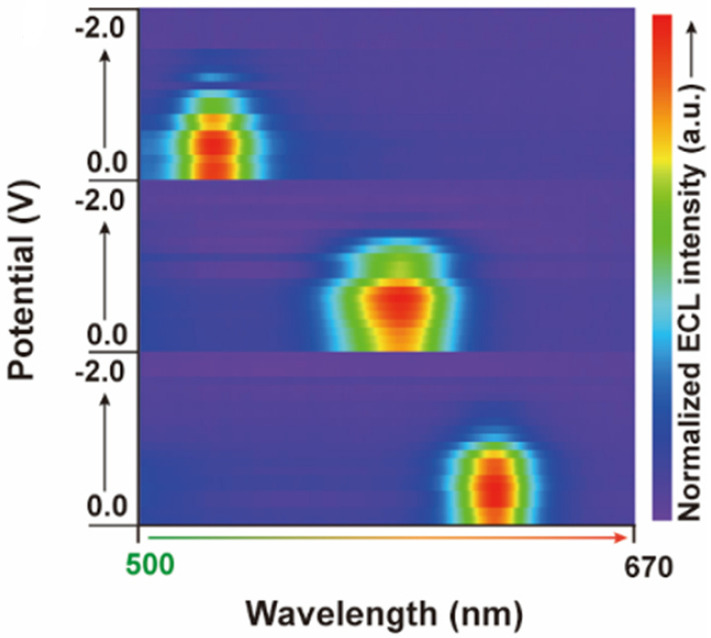
Variation of the color ECL signal relative to the measured potential.

Moreover, a subsequent step regarding the development of the bioimaging system pertains to the construction of color ECL emitters. This considers quantum dots featured by several emission wavelengths. Thus, it was determined that the intensity of the ECL signals varies with the potential. Further visual information in this respect is presented in Figure 3. This suggests that the spectra of the ECL signals generated by the respective emitters were symmetric and narrow. This corresponds to the related fluorescence spectra, while the maximum values of both the ECL and FL spectra were approximately identical. This demonstrates that color ECL emitters may be considered to obtain spectrally proper ECL bioimaging processes. It is relevant to note that the physical features of the related biosensing elements imply that a slightly negative potential is generated, which is natural and does not affect the accuracy of the generated color ECL signal. Additionally, it is important to note that the figure displays the optical evolution of the color ECL signal relative to the measured wavelength interval. Additionally, it is relevant to note that the visualization parameters of the ECL bioimaging setup were optimized to obtain a time-dependent visualization of the biomolecules’ evolution (molecular motion) relative to the membranes of the A549 cells. Thus, the obtained ECL bioimaging setup demonstrated that the number of individual particle entities increased together with a decrease in the measured potential.

### 2.2. Evaluation of the Color ECL Emitters

**Figure 4:**
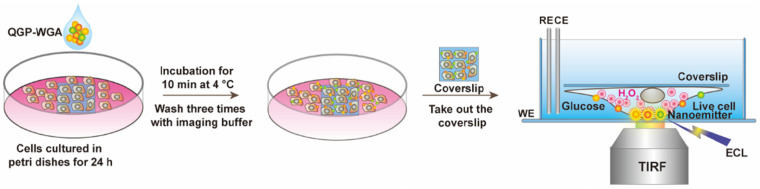
Color ECL bioimaging process, which determines the schematic representation of the ECL imaging model that considers color ECL nano-emitters.

The structural features of the ECL bioimaging construct, particularly regarding the receptors on the cell membrane, are assessed. This evaluation is conducted through the usage of total internal reflection microscopy (TIRF). The bioimaging of molecules on the cell membranes relates to an artificially covered culture environment. Following, the molecules were incubated at a temperature of 5 °C over a period of 17 min. This was followed by a process of inversion that occurred in a customized environment. Further details are visually provided in Figure 4.

Subsequently, fluorescence images were gathered relative to A549 cells. These essentially represent adenocarcinomic human alveolar basal epithelial cells. Thus, A549 cells developed with glucose oxidase enzyme (GOx) proved a consistent absorption of GOx at the level of the cell membrane. Consequently, the GOx was customized using DSPE-PEG2000-NH2 (QGP) produced by Avanti Polar Lipids in Alabaster, Birmingham, AL, USA. Furthermore, the experiments revealed a significantly reduced absorption at the level of the monitored cells’ membrane. Moreover, the customized QGP was further modified using wheat germ agglutinin. The resulting compound, acronymized as QGP-WGA, further enhanced the color ECL signals at the level of the cells’ surface. These specific experimental results demonstrate that QGP may be considered to mark biomolecules to display the biomolecular events, which occur at the level of the cells’ surfaces (membranes).

**Figure 5:**
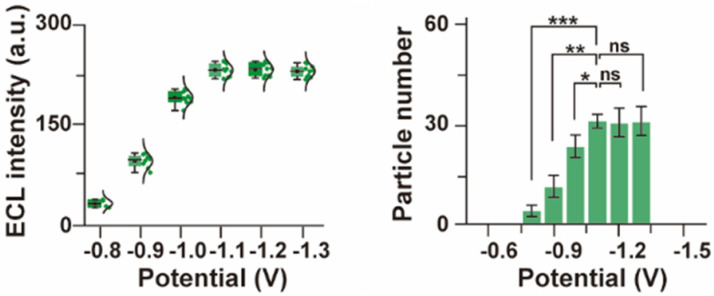
Dynamic variation of the imaged bioparticles relative to the evolution of the applied potential. Here, relative to the potential (*p*), ns means “not significant”, while * is *p* < 0.05, ** is *p* < 0.01, and *** is *p* < 0.001.

The experimental process proceeded with the development of a customized ECL bioimaging system, which considered QGP-WGA compounds. The visualization parameters of the ECL bioimaging setup were optimized to obtain a time-dependent visualization of the biomolecules’ evolution (molecular motion) relative to the membranes of the A549 cells. Thus, the obtained ECL bioimaging setup demonstrated that the number of individual particle entities increased together with a decrease in the measured potential. It is relevant to note that the number of particle entities and their measured intensities did not modify, even after the applied potential was further reduced under −1.1 V. This experimentally observed phenomenon is represented in Figure 5. It is important to recall that the physical features of the related biosensing elements imply that a slightly negative potential is generated, which is natural and does not affect the accuracy of the generated color ECL signal. Consequently, the visual representation in Figure 5 suggests that the gradual decrease of the potential does not negatively influence the quality of the emitted ECL signal. This is an important feature of the proposed visualization process, which differentiates it from similar solutions that are reported in the existing scientific literature.

It is very significant to note that the proposed integrated visualization setup was calibrated considering a thoroughly designed blank control experiment. Thus, the fundamental compound that was used is deionized water. This phase of the experimental calibration process demonstrated that biosensing elements can precisely detect the intended biomolecules and generate accurate color ECL signals, while decalibration has not occurred during the entire experimental process.

The experimental evaluation process also considered the time that was allocated to acquire the images. It was determined that the number of ECL bioparticles and the level of the emitted ECL signals increased proportionally to the time span. Nevertheless, it is relevant to note that an image acquisition time that is greater than one second does not determine any further increase in the reference parameters. Consequently, subsequent experiments were conducted relative to a potential of −1.1 V and an image acquisition time of one second.

Moreover, the experiments considered benign cells, more precisely, Vero and BHK cells. The comparative experimental process considered two variations: QGP-WGA and plain quantum dots WGA (QD-WGA). Thus, the experimental results revealed that the emitted ECL signals were different among the three types of analyzed cells: A549, Vero, and BHK. The explanation for this difference resides in the higher content of hydrogen peroxide in malign cells (A549) compared to the benign cells (Vero and BHK).

**Figure 6:**
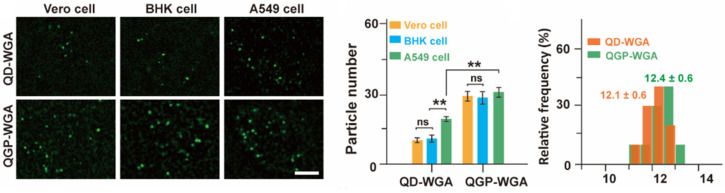
Comparative analysis relative to benign cells, which includes a representation of the monitored ECL signals. Here, considering the potential (*p*), ns means “not significant”, while ** is *p* < 0.01.

Furthermore, the experiments that were conducted proved that relative to the QGP-WGA variation, the variability of the emitted ECL signals is substantially reduced among the considered cell types. Relevant images, which were produced by the experimental process, are presented in Figure 6. Interestingly, the experimental outcomes established variations in the quality of the bioimaging visualizations, which pertain to the intensity of the emitted ECL signals. Thus, the ECL signals were consistently intense relative to the QD-WGA variation for the A549 cells type but significantly decreased as soon as the hydrogen peroxide was applied. Conversely, no significant variation of the emitted ECL signals was noted relative to the QGP-WGA variation, even after the addition of hydrogen peroxide. Consequently, it is relevant to note that the QGP-WGA variation produced consistently bright and structurally stable visualization frames of the reference cells and biomolecules, regardless of the hydrogen peroxide presence or absence. This clearly suggests that QGP can withstand adverse environmental factors and constantly generate high-quality visualization frames of the target cell surfaces.

**Figure 7:**
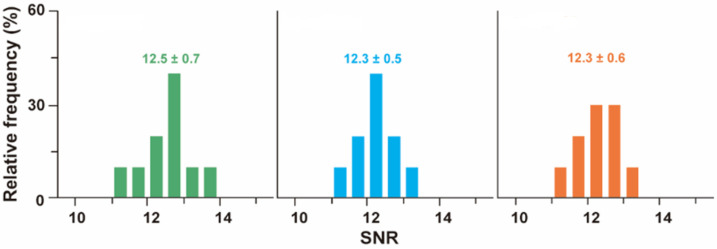
Comparison of TIP bioimaging processes relative to the reference wavelengths of 540 nm, 585 nm, and 630 nm. The green bars correspond to the wavelength of 540 nm, while the blue and orange bars correspond to 585 nm and 630 nm, respectively.

Furthermore, the emission of color ECL signals was evaluated relative to biomolecules that belong to the surface of the A549 malign cells. Thus, the color TIP bioimaging processes considered several wavelengths during the experimental process, more precisely 540 nm (QGP540), 585 nm (QGP585), and 630 nm (QGP630). Thus, signal-to-noise measurements were conducted, which proved that this visualization approach determines intense ECL signals and a low amount of background noise in the generated image frames. This result is particularly relevant for the proper generation of color ECL signals. Further details are visually presented in Figure 7, which displays the varieties QGP540, QGP585, and QGP630 in green, blue, and orange, respectively. It is important to note that the graphs that are displayed in Figure 7 essentially describe the correlation between the relative frequency and the measured signal-to-noise ratio. Therefore, considering the direct nature of the correlation, we omitted the addition of an error bar to each of the graphs’ bars.

### 2.3. Practical Relevance of Quantum Dots

**Figure 8:**
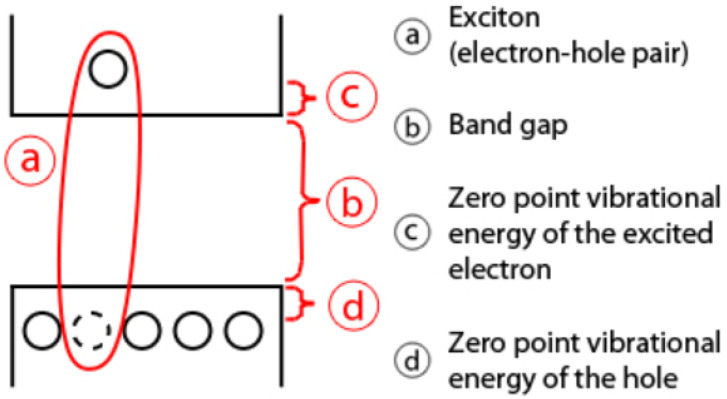
Optical properties and mechanics of quantum dots.

Relative to semiconductors, the absorption of light generally relates to the excitement of an electron, considering the valence of the conduction band, which leaves behind a hole. Thus, the electron and the hole can bind to each other to generate an exciton. As soon as this exciton recombines, the exciton’s energy can be transformed into emitted light. The determined phenomenon is known as fluorescence. Essentially, the energy of the emitted photon may be regarded as the sum of the band gap energy between the highest occupied level, the lowest unoccupied energy level, the confinement energies of the hole and the excited electron, and also the bound energy of the exciton, which essentially determines the electron–hole pair. The process is visually described in Figure 8.

Quantum dots are especially relevant for optical applications, considering their high extinction coefficient and very fast optical nonlinearities. This suggests their possible inclusion in real-world applications that concern all-optical systems, like the integrated monitoring and visualization model that is proposed. Quantum dots function like a single-electron transistor. Interestingly, they have also been suggested as foundational elements for the implementation of qubits, which are fundamental constituent and processing elements of quantum computers.

Considering the zero-dimensional nature of quantum dots, they feature a superior density of states than higher-dimensional structures. Therefore, they provide superior transport and optical properties. Consequently, they are compatible with real-world use case scenarios, such as diode lasers, amplifiers, and biological sensors like the ones that are described in this paper. High-quality quantum dots are proper for optical encoding and multiplexing systems, considering their broad excitation profiles and also the narrow and symmetric emission spectra. The recent versions of quantum dots feature greater potential relative to the study of intracellular processes at the level of individual molecules, which determines high-resolution cellular imaging processes and long-term in vivo observation of cell dynamics. Therefore, it is immediate to observe that quantum dots appear as a natural solution for the design and implementation of relevant TIP tracking elements that are presented in this paper.

## 3. Results and Discussion

### 3.1. Remarks Concerning the Monitoring of Molecular Motion

The implementation of TIP molecular imaging processes requires the usage of special microscopes, which are considered to gather raw image data of the target molecular particles. This also supports the acquisition of data regarding the dynamic time-dependent evolution of the reference molecules and cells.

**Figure 9:**
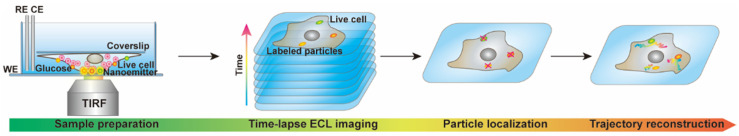
Logical workflow of the TIP molecular imaging process, which relates to a schematic representation of the color ECL TIP tracking process.

Thus, we have conducted extensive experiments concerning the generation of ECL images related to the cell’s surface, considering various time series. The logical and functional structure of the related bioimaging process is presented in Figure 9. Initially, a continuous stream of image frames, which is related to both the QD-WGA and QGP-WGA variations, was generated using the A549 cells. The outcomes of this experimental phase suggest that the emitted ECL QD-WGA signals at the level of the cell surface progressively reduced their intensity, owing to the continuous addition of hydrogen peroxide. Conversely, the emitted ECL QGP-WGA signal followed a stable pattern over time, which fully confirms the outcomes of the preceding experimental stages. It is also relevant to note that the number of observed bioparticles did not vary significantly.

Therefore, it is natural to observe that the QGP-based variations feature the necessary characteristics to implement the required real-time ECL bioimaging. Consequently, we have selected this technical solution to implement the necessary TIP tracking processes. The individual reference points in the acquired images were isolated using a 2D Gaussian fitting method. Furthermore, the reconstruction of the bioparticles’ paths on the cells’ membranes is realized by considering proper links between the identified points in each image frame. The experimental analysis also demonstrated that the particles of wheat germ agglutinin remain long enough on the cell surface for the entire set of experiments to properly unfold.

### 3.2. Evaluation of Intermolecular Interactions Using Proposed TIP Tracking Approach

The described TIP tracking approach was also considered to evaluate the interactions that manifest between sphingomyelin, cholesterol, and wheat germ agglutinin. Sphingomyelin and cholesterol are fundamental lipids that are found in the mammalian cell surfaces. They can produce lipid formations in the plasma structure of the cell surface. Therefore, they can act functionally at the level of the cell. The scientific literature that was reviewed suggests that a combination between lysenin and the D4 domain of Perfringolysin O (PFO D4) may be considered to detect sphingomyelin and cholesterol [31,32].

**Figure 10:**
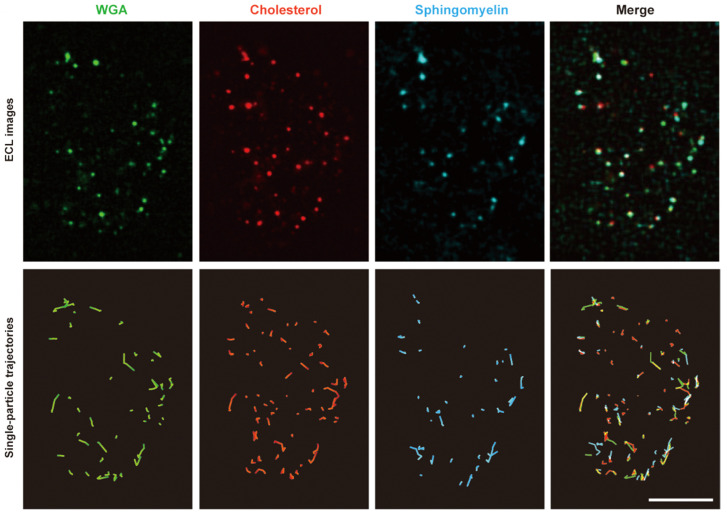
Evaluation of dynamic interactions established between sphingomyelin, cholesterol, and WGA.

Consequently, the dynamic interactions established between sphingomyelin, cholesterol, and WGA were studied considering a color ECL TIP cellular tracking process. Essentially, the technical solution involves that color ECL emitters are used to properly mark PFO D4, lysenin and WGA. Certain images that were obtained during this phase of the experimental evaluation process are presented in Figure 10. Thus, it can be observed that the WGA signals were detected on the zones of the cellular surface that contained sphingomyelin, cholesterol, or both. It is interesting to note that the cohesion of these formations was preserved even when certain motions were applied. Nevertheless, the experiments revealed that the local cohesion between WGA and sphingomyelin is less reliable than the local cohesion of formations made of WGA and cholesterol.

**Figure 11:**
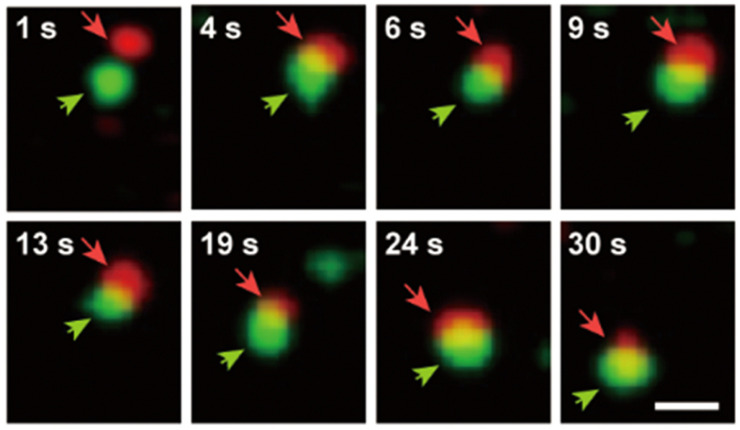
Time-dependent dynamic interaction between cholesterol and WGA. The cholesterol molecules are represented with green, while WGA is determined by the red component. The reference scale is 2 μm.

Furthermore, we have effectively used the proposed ECL TIP cellular tracking process to obtain a color set of images that depict the interactions between WGA and cholesterol. Thus, the generated paths clearly showed the time-dependent dynamic interaction between cholesterol and WGA at the level of the cell’s surface. More precisely, Figure 11 presents some of the obtained image framesconsidering a scale of 2 μm. The outcomes of the experimental evaluation process, which were described, clearly demonstrate that the proposed visualization model achieves the necessary color ECL TIP tracking capabilities. Therefore, it can be used to implement a systematic dynamic monitoring of the interactions that are established between biomolecules, which manifest on the cell membranes. It is also relevant to note that Figure 11 visually represents the time-dependent evolution of the interaction between cholesterol and WGA considering eight time intervals. This suggests that the visualization system can be configured to generate snapshots at various time intervals, which will produce a more accurate time-dependent representation of the target biomolecular interactions.

### 3.3. Contextual Overview Concerning the Merits of Reported Contribution

Naturally, the surveyed scientific literature reported contributions concerning the bioimaging of cellular and molecular structures. The following paragraphs consider the most promising and computationally efficient solutions that were identified during the literature review stage of the presented research process.

Thus, papers [4,5,6,7,8,9] described visualization techniques that are based on the utilization of certain biochemical compounds. Nevertheless, the reported experimental outcomes suggest that the obtained images are not of sufficient resolution and sharpness, while the color details are either impossible to render or inaccurately displayed.

Article [10] reported a bioimaging technique that considers an optically modulated fluorescence solution. In our laboratory, we replicated the reported steps, but the obtained visualizations lacked the required color variants. Furthermore, paper [13] described a visualization model that concerns the electrochemiluminescence microimaging of cell membrane proteins. The experimental replication steps that we conducted suggest that the solution produces low-resolution images that cannot be reliably considered to generate time-dependent molecular paths. Consequently, it is essentially impossible to define the monitored biomolecules’ dynamic evolution. Furthermore, papers [14,15,16,17,18,19] proposed contributions with similar drawbacks.

It is relevant to note that article [20] described one of the most promising approaches that we have reviewed and tested in our laboratory. Thus, the related approach determines the generation of relatively stable image frames over time, while the color details are rendered considering a sufficient level of accuracy. Nevertheless, the obtained levels of detail and sharpness are not sufficient to properly highlight the time-dependent paths of the monitored biomolecules. Additionally, similar drawbacks were observed during the laboratory evaluation of the solution that was proposed in article [21].

It is important to note that the rigorous assessment of the mentioned solutions considered both the experimental results reported by the surveyed papers, and also the experimental evaluation that was conducted in our laboratory. Consequently, it is immediate to note that the existing approaches that were analyzed do not fulfill all the required constraints. Therefore, the fully functional solution that is reported in this article possesses the following unique features.

- It considers readily available organic, inorganic, electrical, and electronic components to design and implement the required infrastructure.
- It can accurately detect the motion of biomolecules on the target cells’ surface.
- Consequently, it precisely monitors the time-dependent evolution of the biomolecules that exist on the target cells’ surface.
- It generates consistently sharp and clear image frames over time.
- It can produce color images while the respective color details are rendered precisely in accordance with their biomolecular role.
- The generated image frames’ resolution is sufficient to precisely capture the motion of the analyzed biomolecules.
- Consequently, it is possible to accurately represent the paths of the observed biomolecules on the surface of the respective cells.

Therefore, it is immediate to note that the approach, which this paper reports is, to the best of our knowledge, one of the few that fulfills these fundamental constraints and requirements.

## 4. Conclusions

The continuous, reliable monitoring and visualization of biomolecules that are part of cell membranes has traditionally constituted a challenge. The relevant scientific literature reports solutions that either do not allow the implementation of fully reliable real-time monitoring systems or struggle to produce the required color images. In this context, it may be asserted that the results of the research process, which this paper reports, established that electrochemiluminescence biosensors assemblies offer significant advantages regarding the involved microscopic imaging processes. Consequently, this paper described and analyzed a quantum dots-based biosensor assembly, which is used to implement a fully functional color ECL visualization system that allows for cellular and biomolecular structures to be accurately visualized. The related nano-emitter allows the implementation of real-time bioimaging scenarios. Consequently, the proposed approach is thoroughly evaluated relative to the time-dependent evolution of biomolecules. It has been demonstrated that traditionally problematic structures, like the biomolecules that are part of cell membranes, can be studied and monitored relative to their time-dependent dynamic evolution using the proposed solution. The reported research process has been conducted in the realm of cooperation with a specialized biomedical engineering company, and the presented results are expected to substantially support a better understanding of the biomolecules’ time-dependent dynamic evolution.

## Data Availability

Data is contained within the article.
